# Supplementary material for: p53 orchestrates DNA replication restart homeostasis by suppressing mutagenic RAD52 and POLθ pathways
Source: eLife. 2018 Jan 15;7:e31723. doi: 10.7554/eLife.31723 (PMC5832412; doi:10.7554/eLife.31723)
Supplement: Supplementary file 1. [file elife-31723-supp1.docx]

**'Supplementary File 1.**

| **Figure** | **cell line** | **mean EdU-PLA** | **Variance EdU-PLA** | **SIRF** | **SIRF mean** | **SIRF variance** | **Z-score** | **p-value** |
| --- | --- | --- | --- | --- | --- | --- | --- | --- |
| 3C | HAP-1 WT | 342.04 | 12743.72 | MLL3 | 10.86 | 16.82 | -0.52 | 0.302 |
|  | HAP-1 p53 KO | 361.66 | 29142.89 | MLL3 | 11.87 | 28.12 |  |  |
| 3D | HAP-1 WT | 444.28 | 45895.02 | MLL3+HU | 17.38 | 84.03 | -4.23 | <0.0001 |
|  | HAP-1 p53 KO | 513.29 | 53337.60 | MLL3+HU | 13.11 | 31.92 |  |  |
| 3E | HAP-1 WT | 444.28 | 45895.02 | MRE11+HU | 29.74 | 860.04 | -2.61 | 0.004 |
|  | HAP-1 p53 KO | 513.29 | 53337.60 | MRE11+HU | 18.28 | 358.82 |  |  |
| Supp. 3 | HAP-1 WT | 342.04 | 12743.72 | MRE11 | 18.93 | 146.50 | -0.66 | 0.255 |
|  | HAP-1 p53 KO | 513.29 | 53337.60 | MRE11 | 18.28 | 358.82 |  |  |
| 4A | HAP-1 WT | 943.93 | 75378.36 | RAD51+HU | 23.70 | 197.37 | 1.74 | 0.0409 |
|  | HAP-1 p53 KO | 866.58 | 89865.89 | RAD51+HU | 18.62 | 184.76 |  |  |
| 4B | Saos-2 | 1156.03 | 415114.06 | RAD51+HU | 87.56 | 4279.58 | U2OS vs Saos-2: -2.8 | 0.0026 |
|  | U2OS | 1619.89 | 248134.72 | RAD51+HU | 16.90 | 147.32 | U2OS vs R175H: 7.01 | <0.0001 |
|  | Saos-2 R175H | 727.39 | 317248.35 | RAD51+HU | 23.77 | 158.64 | U2OS vs R273H: 7.09 | <0.0001 |
|  | Saos-2 R273H | 712.52 | 177220.96 | RAD51+HU | 17.58 | 95.91 |  |  |
| 4C | HAP-1 WT | 492.97 | 93235.94 | RAD52+HU | 493.63 | 28382.91 | 7.11 | <0.0001 |
|  | HAP-1 p53 KO | 430.33 | 49005.89 | RAD52+HU | 762.44 | 171658.31 |  |  |
| 4D | HCT116 WT | 508.14 | 21611.71 | RAD52+HU | 19.92 | 87.11 | 5.97 | <0.0001 |
|  | HCT116 GOF R248W | 359.54 | 6267.23 | RAD52+HU | 42.67 | 188.06 |  |  |
| Supp. 4A | HCT116 WT | 508.14 | 21611.71 | RAD51+HU | 11.61 | 35.55 | 6.14 | 0.00017 |
|  | HCT116 GOF R248W | 359.54 | 6267.23 | RAD51+HU | 39.27 | 356.99 |  |  |
| Supp. 4B | H1299 WT | 442.22 | 111386.43 | RAD51+HU | 17.07 | 57.40 | 3.58 | <0.0001 |
|  | H1299 p53 S47 | 356.53 | 28829.41 | RAD51+HU | 34.88 | 415.03 |  |  |
| Supp. 4D | Saos-2 | 1156.03 | 415114.06 | RAD52+HU | 17.00 | 80.60 | U2OS vs Saos-2: -2.46 | 0.007 |
|  | U2OS | 1619.89 | 248134.72 | RAD52+HU | 12.27 | 47.18 | U2OS vs R175H: 7.01 | <0.0001 |
|  | Saos-2 R175H | 727.39 | 317248.35 | RAD52+HU | 18.41 | 161.07 | U2OS vs R273H: 7.14 | <0.0001 |
|  | Saos-2 R273H | 712.52 | 177220.96 | RAD52+HU | 19.60 | 204.24 |  |  |
| Supp. 4E | H1299 WT | 442.22 | 111386.43 | RAD52+HU | 26.94 | 308.53 | 3.92 | <0.0001 |
|  | H1299 p53 S47 | 356.53 | 28829.41 | RAD52+HU | 60.32 | 1768.95 |  |  |
| 5B | HAP-1 WT | 610.76 | 29741.77 | POLQ+HU | 5.71 | 10.59 | HAP-1 vs p53 null: 5.97 | <0.0001 |
|  | HAP-1 p53 KO | 506.04 | 14924.29 | POLQ+HU | 8.15 | 28.52 | p53 null vs HAP-1+PFM39: -0.50 | 0.309 |
|  | HAP-1 WT +PFM39 | 522.96 | 16834.56 | POLQ+HU | 8.31 | 28.02 | HAP-1 vs HAP-1+PFM39: 2.74 | 0.003 |
